# Agreement between visual inspection and objective analysis methods: A replication and extension

**DOI:** 10.1002/jaba.921

**Published:** 2022-04-27

**Authors:** Tessa Taylor, Marc J. Lanovaz

**Affiliations:** ^1^ University of Canterbury/Te Whare Wānanga o Waitaha; ^2^ Paediatric Feeding International; ^3^ Université de Montréal

**Keywords:** artificial intelligence, conservative dual criteria, interrater agreement, machine learning, visual inspection

## Abstract

Behavior analysts typically rely on visual inspection of single‐case experimental designs to make treatment decisions. However, visual inspection is subjective, which has led to the development of supplemental objective methods such as the conservative dual‐criteria method. To replicate and extend a study conducted by Wolfe et al. (2018) on the topic, we examined agreement between the visual inspection of five raters, the conservative dual‐criteria method, and a machine‐learning algorithm (i.e., the support vector classifier) on 198 AB graphs extracted from clinical data. The results indicated that average agreement between the 3 methods was generally consistent. Mean interrater agreement was 84%, whereas raters agreed with the conservative dual‐criteria method and the support vector classifier on 84% and 85% of graphs, respectively. Our results indicate that both objective methods produce results consistent with visual inspection, which may support their future use.

Visual inspection is commonly used to evaluate the results of single‐case experimental designs. Although some researchers have reported positive findings (Ford et al., 2020; Kahng et al., 2010; Novotny et al., 2014), many studies have questioned the reliability of visual inspection for identifying behavior change in single‐case graphs (Dart & Radley, 2017; DeProspero & Cohen, 1979; Ninci et al., 2015; Wilbert et al., 2021; Wolfe et al., 2016, 2018). Therefore, researchers have proposed different supplemental methods to analyze single‐case data more objectively (Fisher et al., 2003; Krueger et al., 2013; Lanovaz et al., 2020; Manolov & Vannest, 2019). Objective methods aim to complement rather than replace visual inspection to improve reliability, validity, and decision making; decrease errors; assist with training efficiency; improve communicability of results; and provide quantitative data on the treatment effect. Notably, Fisher et al. (2003) developed the dual‐criteria and conservative dual‐criteria methods, which have been the topic of a growing number of studies examining their validity (Falligant et al., 2020; Lanovaz et al., 2020; Lanovaz et al., 2017; Wolfe et al., 2018). Although researchers have shown that these methods can adequately control Type I error rates (e.g., Falligant et al., 2020; Lanovaz et al., 2017), studies have also noted that their power could be improved (Fisher et al., 2003; Manolov & Vannest, 2019).

In a recent study on the topic, Wolfe et al. (2018) evaluated agreement between visual inspection and the conservative dual‐criteria method with 31 multiple baseline graphs published in peer‐reviewed journals. The visual inspection involved 52 expert raters who had authored at least five studies that relied on single‐case methodology. Expert raters had to categorize whether there was a change in the dependent variable for each panel and whether the graph showed a functional relation. The researchers found a mean agreement between expert raters of 83% and an agreement of 84% between the raters and the conservative dual‐criteria method. However, one of the limitations of the study was that the analyses relied on published data. Published data may differ considerably from clinical data (e.g., less stability), which is why replication may be important (Dowdy et al., 2020; Sham & Smith, 2014).

Recently, some researchers have proposed using machine learning as an objective supplement to visual inspection to analyze single‐case graphs (Lanovaz et al., 2020). Given that most behavior analysts do not have prior knowledge or training on machine‐learning algorithms, we will introduce the topic before explaining the purpose of our study. At its broadest, machine learning involves the use of computer algorithms^1^ to detect and use patterns in data. These problems can take on many forms, but Lanovaz et al. (2020) focused on binary classification problems. A binary classification problem has only two possible outcomes—true or false. In Lanovaz et al., this value represented whether a graph showed a clear change (true) or not (false). To conduct classification, the algorithms also need input data on which to make their decisions. Lanovaz et al. used the means, standard deviations, intercepts, and slopes of Phases A and B as input data.

In supervised machine learning, the experimenter provides the input and outcome data to algorithms, which train the models to make predictions. Specifically, the algorithms transform the input data to develop a model that can predict the outcome data. Each algorithm attempts to optimize correct predictions by transforming the data in a different way. The ultimate test of the appropriateness of responding by a model involves comparing the predictions with the correct labels on data that have never been used during training (i.e., generalization). Many different types of algorithms exist to train these models. The paragraph below briefly describes the algorithm that we will test as part of the current study—the support vector classifier.

The support vector classifier uses a function to project the data in a higher dimension and optimizes the split between the two categories. Figure 1 shows a simple example of how a support vector classifier may split data. The upper panel shows a binary outcome (represented by opened and closed data points) that cannot be separated using traditional linear regression. The lower panel shows how projecting the data in a higher dimension allows the data to be separated by a plane (referred to as a hyperplane in higher dimensions). The support vector classifier can make predictions by examining where a novel data point falls within this higher dimension. In a comparison to visual inspection, Lanovaz and Hranchuk (2021) trained models including the previous algorithm to identify changes in single‐case AB graphs. Their results showed that the support vector classifier produced lower Type I error rates (fewer false positives) and higher power (fewer false negatives) than the conservative dual‐criteria method and visual raters on simulated data. Moreover, the support vector classifier generally agreed more often with visual raters than the conservative dual‐criteria method. However, extensions of Lanovaz and Hranchuk remain necessary as the study focused on simulated data, which may differ from their nonsimulated counterparts. Examining correspondence with visual raters on actual, nonsimulated graphs appears important.

**Figure 1:**
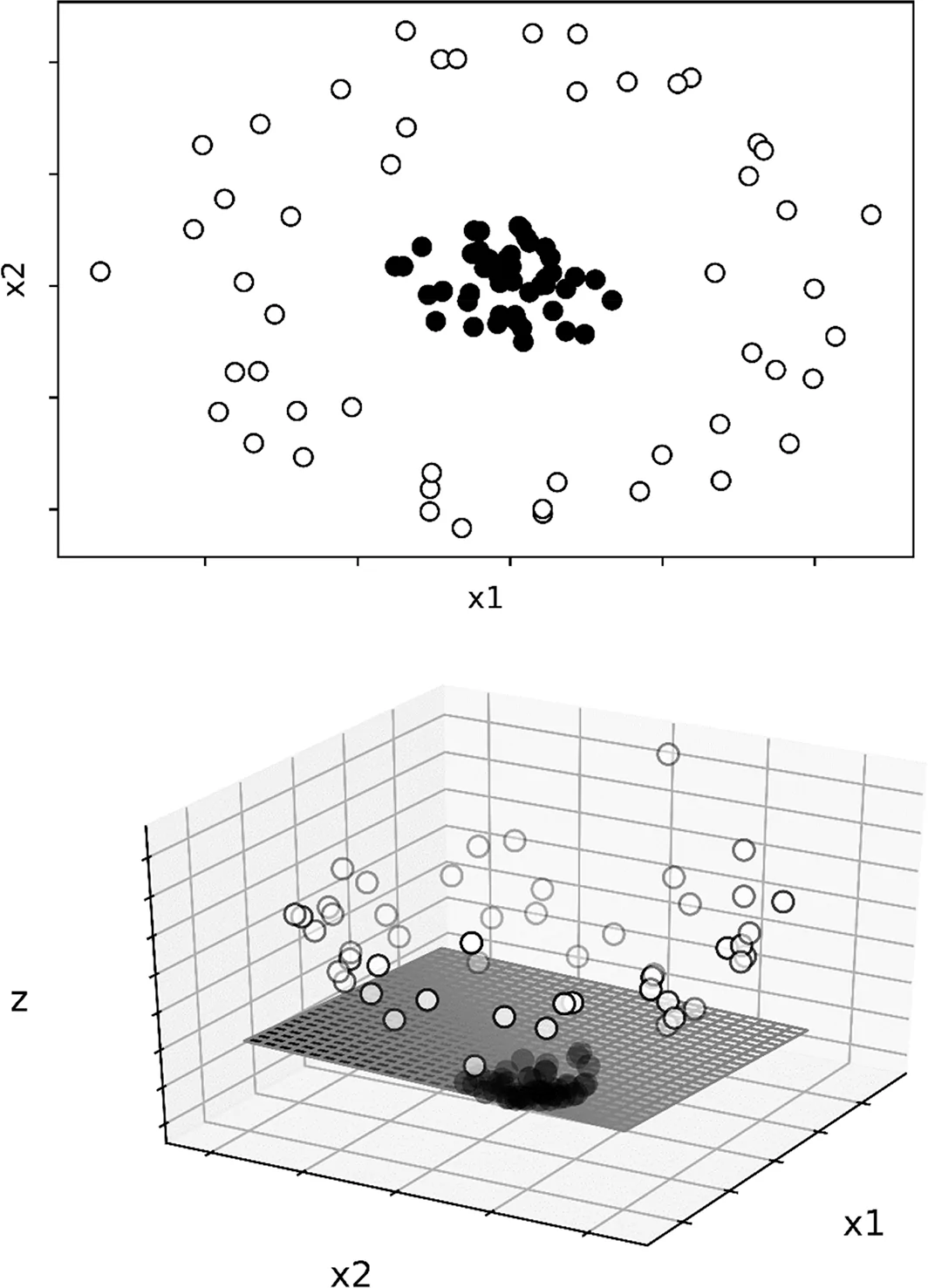
Example of a Dataset Separated by a Support Vector Classifier *Note*. The upper panel shows a two‐dimensional graph representing two features: x1 and x2. Closed points represent one category and opened points represent a different category. The lower panel depicts the addition of a higher dimension (z) and a linear plane that separates the two categories. Reprinted with permission from “Machine Learning to Analyze Single‐Case Data: A Proof of Concept” by M. J. Lanovaz, A. R. Giannakakos, and O. Destras, 2020, *Perspectives on Behavior Science* (https://doi.org/10.1007/s40614‐020‐00244‐0). CC BY 4.0.

The purpose of our study was to replicate and extend Wolfe et al. (2018) by examining agreement between visual inspection and the conservative dual‐criteria method. We extended Wolfe et al. by using a larger number of graphs to examine within‐subject error, blinded normalized graphs, and modified reversal/withdrawal designs. A secondary purpose was to replicate and extend Lanovaz and Hranchuk (2021) by examining correspondence between visual inspection, the conservative dual‐criteria method, and the machine‐learning algorithms on a novel dataset. We extended both studies by using clinical data and adding a 10‐point scale with definitional criteria to visual‐inspection ratings.

## Method

### Data Acquisition

The sample consisted of all clinical cases from an intensive, in‐home pediatric feeding treatment program in Australia. The data were not previously published in a prior consecutive controlled case series (Taylor et al., 2021) and each case included a treatment evaluation conducted via single‐case experimental design (*N* = 6). All treatment evaluations used modified withdrawal/reversal designs: ABCAC (*n* = 2), ABCDEAE (*n* = 3), and ABCDEFAF (*n* = 1). The parents consented to their child's data being used for research and this research project was approved by the second author's university research ethics board.

The six dependent variables were: clean mouth percentage (percentage of trials in which the mouth was clean of food/liquid; permanent product measure of swallowing/consumption), latency to clean mouth, latency to acceptance (bite/drink enters the mouth), inappropriate mealtime behavior per minute (head turn, mouth cover, pushing feeder away), expulsion per minute (bite/drink exits mouth), and negative‐vocalizations percentage (percentage of session duration with crying or negative statements about the meal). The expected direction of the treatment effect was an increase in clean mouth (i.e., food consumption/swallowing) and a decrease in the remaining variables. We used data from the complete phases and compared each adjacent phase. For example, one ABCAC treatment evaluation yielded four phase comparisons (A1B1, B1C1, C1A2, A2C2) for each of the six variables, producing 24 total phase comparisons for this participant. These phase comparisons were then graphed separately (i.e., distinct AB graphs for each variable). This process yielded 198 AB phase comparisons for the six participants (i.e., 33 adjacent phases with six distinct variables per comparison). The data and code are available in online repository at: https://osf.io/2wgtu/.

To construct the 198 graphs, we used Python (Version 3.7.7) to standardize the process. The graph title provided only the expected direction of the change (i.e., ‐1.0 for decrease and 1.0 for increase), and the vertical axis was labelled generically as “Behavior” with unlabeled tick marks. We removed the values beside the tick marks to standardize the presentation of the graphs and to control for the effects of differing axis values on the analysis of visual raters. As such, the raters had to focus on the relative change from one phase to another to categorize each graph. A phase line separated Phases A and B, and the horizontal axis was labelled, “Session” with the numerical values depicted on the tick marks. These graphs were blinded in that they did not provide the variable label on the vertical axis, the scale of the vertical axis, phase labels or design sequence letters (e.g., ABCAC), or participant information (see graphsforanalysis.pdf in online repository).

### Visual Inspection and Interrater Agreement

For visual inspection of each phase comparison (*N* = 198), raters responded “yes” or “no” to the following question: “Would the change observed from one phase to the next be indicative of functional control of the behaviour in the planned direction (i.e., increase or decrease) if it were reversed and replicated?” Raters also provided a continuous value from 0 (certainty of no effect in the planned direction) to 10 (certainty of an effect in the planned direction), with 0 to 4 corresponding to “No” and 5 to 10 corresponding to “Yes” (Taylor & Lanovaz, 2021). Five doctoral‐level behavior analysts with PhDs in psychology (one professor, four practitioners) made ratings based on the blinded AB graphs (see ExpertA.xlsx, ExpertB.xlsx, ExpertC.xlsx, ExpertD.xlsx, and ExpertE.xlsx for complete analyses). The raters had trained at three different universities and were over 10 years postgraduation. Four raters completed pre‐ and/or postdoctoral training at the same internship and fellowship program and were licensed psychologists. All raters had authored peer‐reviewed research publications, and three raters had authored at least five single‐case experimental design publications. Four raters were board‐certified behavior analysts, and one was the first author.

### Conservative Dual‐Criteria Method

To remain consistent with Wolfe et al. (2018), we used the conservative dual‐criteria method (Fisher et al., 2003). This method involves projecting trend and mean lines from baseline onto the next phase, adjusting the lines by 0.25 standard deviations from the baseline data, counting the number of data points above or below both lines depending on the expected direction of change, and comparing the results to a cut‐off value based on the binomial distribution. Our python code repeatedly applied this analysis to each AB comparison (see CDC_analysis.py for code and CDC_Results.csv for results of the analysis).

### Machine Learning

For each phase comparison, our code applied a model derived from machine learning to determine whether procedures produced a clear change in the expected direction (see ML_analysis.py for code and ML_Results.py for results of the analysis). Specifically, our analyses involved applying the support vector classifier previously described and developed by Lanovaz and Hranchuk (2021). We selected the support vector classifier because it produced the fewest errors during the analyses. Furthermore, the support vector classifier agreed more frequently with expert behavior analysts than the behavior analysts amongst themselves in a study with simulated data (Lanovaz & Hranchuk, 2021). The support vector classifier used the eight features extracted from the standardized data (mean, standard deviation, intercept, and slope of each phase) and provided output decisions based on the probability of a clear change in the expected direction. Each of these features represented important characteristics of the data used during visual inspection: Mean is related to level change, standard deviation to variability, intercept to immediacy of change, and slope to trend (Lanovaz & Hranchuk, 2021). Probabilities above, or equal to, 0.5 were categorized as a clear change.

As an example of applying this method, assume that we want to categorize a graph with five points in Phase A and seven points in Phase B using a support vector classifier. The first step involves extracting the eight features from our graph. To do so, we transform each data point to a *z* score to normalize the data by subtracting the mean of the graph from the value of each point and dividing this difference by the standard deviation for the graph. If the purpose of the interventions is to reduce behavior, the *z* scores must also be multiplied by ‐1. Once the points have been standardized, the code uses the *z* scores to extract the mean, standard deviation, intercept, and slope for each phase (eight features). The second step involves providing these eight features to the model previously developed by Lanovaz and Hranchuk (2021). The model transforms the features by adding an extra dimension and places the data for our graph in a multidimensional space (as depicted in three dimensions in Figure 1). The model also has a hyperplane, which separates the multidimensional space in two (no change vs. change). The categorization then depends on the position of the multidimensional value relative to this hyperplane. If we take the exemplar presented in Figure 1 (bottom panel), each graph would produce a single multidimensional point that falls either above or below the plane (determining its category).

### Analyses

For each rater and method, we calculated a pairwise percentage of agreement by dividing the number of agreements (i.e., change vs. no change) by the total number of graphs (see Comparison.py). We also calculated Cohen's kappa (Cohen, 1960). Kappa values range from ‐1 (perfect disagreement) to 1 (perfect agreement), with 0 indicating completely random agreement. Landis and Koch (1977) proposed interpretive guidelines of slight agreement (0–0.20), fair agreement (0.21–0.40), moderate agreement (0.41–0.60), substantial agreement (0.61–0.80) and almost perfect agreement (0.81–1.0). For continuous outcomes, our code computed a Spearman's rho pairwise correlation between visual‐inspection ratings and machine‐learning probability values for each graph. The conservative dual‐criteria method was excluded from the prior analysis because it does not produce a probability value. For each rater and method, we compared average agreement separately based on whether the conservative dual‐criteria method and the support vector classifier indicated an effect or no effect.

The next step involved a more in‐depth analysis of patterns of disagreements between the visual raters, the conservative dual‐criteria method, and the support vector classifier (see disagreement_analysis.py). First, we created four groups of graphs. The first group of graphs, agreement on visual inspection, included only the graphs for which at least four of five raters agreed on the outcome (*n* = 175). The second group of graphs, disagreement on visual inspection, involved the remaining graphs for which only three raters agreed (*n* = 23). The next two groups were subsets of the graphs showing agreement on visual inspection. The third group included graphs on which the conservative dual‐criteria method disagreed with the visual raters in cases where four or five raters agreed (*n* = 15). Similarly, the final group involved graphs with visual agreement with which the support vector classifier disagreed (*n* = 13). Finally, we compared the agreements and disagreement across different lengths of Phases A and B.

## Results

Proportion of correspondence for the binary outcomes are presented in Table 1. Interrater agreement using visual inspection averaged 84.3% (range, 72%–91%) across all raters with kappa of .66 (range, .43–.79). The support vector classifier matched the ratings of the behavior analysts on 85.0% (range, 78%–89%) of graphs with kappa of .67 (range, .55–.75), on average. The conservative dual‐criteria method averaged 83.6% (range, 79%–87%) correspondence with visual‐inspection ratings with kappa of .64 (range, .58–.72). The support vector classifier corresponded 81.0% with the conservative dual‐criteria method with kappa of .59. Table 2 presents the correlations for the continuous outcomes. The correlation coefficient for visual inspection averaged .78 (range, .66–.90) across all raters. In comparison, the support vector classifier had an average correlation coefficient of .74 (range, .60–.79) with the visual raters.

**Table 1:** Proportion of Correspondence and Kappa Agreement Between the Different Methods (Binary Outcomes)

	Expert A	Expert B	Expert C	Expert D	Expert E	CDC Method
Expert A						
Expert B	.79 / .57					
Expert C	.80 / .59	.90 / .77				
Expert D	.72 / .43	.88 / .71	.86 / .67			
Expert E	.83 / .65	.91 / .79	.89 / .75	.85 / .65		
CDC Method	.79 / .58	.84 / .64	.87 /.72	.83 / .59	.85 / .67	
SVC	.78 / .55	.89/ .75	.87 / .71	.84 / .63	.87 / .71	.81 / .59

*Note*. For each pair, the proportion of correspondence is on the left of the slash and the kappa value on the right. CDC: conservative dual‐criteria, SVC: support vector classifier.

**Table 2:** Correlation Between the Different Methods (Continuous Outcomes)

	Expert A	Expert B	Expert C	Expert D	Expert E
Expert A					
Expert B	.66				
Expert C	.66	.86			
Expert D	.66	.82	.84		
Expert E	.72	.90	.87	.82	
SVC	.60	.79	.76	.78	.76

*Note*. SVC: support vector classifier.

**Figure 2:**
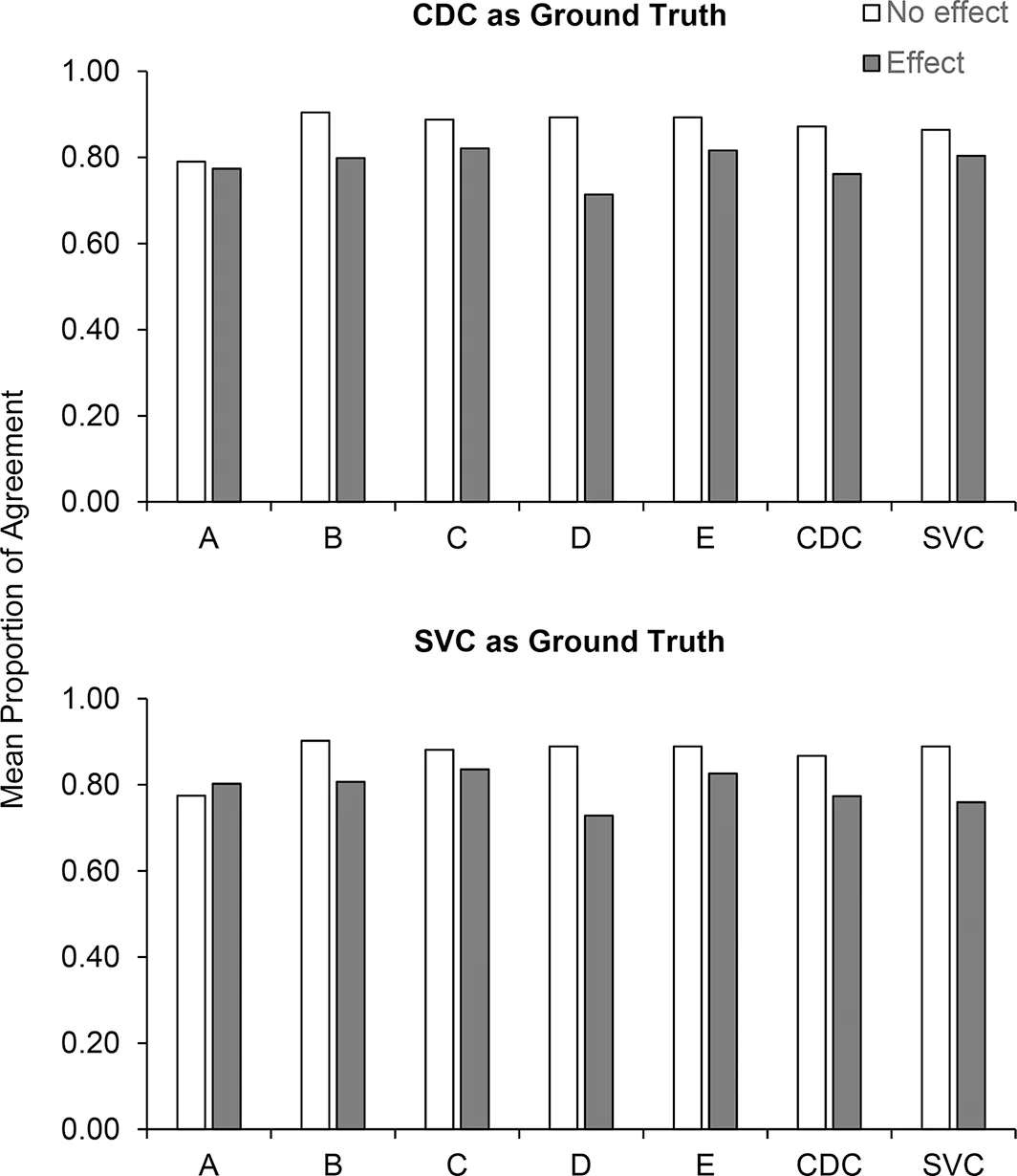
Average Agreement of Each Analysis When the Conservative Dual‐Criteria (CDC) and Support Vector Classifier (SVC) Indicated an Effect or No Effect

Figure 2 presents average agreement based on results from the conservative dual‐criteria (top panel) or support vector classifier (bottom panel) indicating an effect or no effect. The support vector classifier found an effect in 35.4% of graphs, and the conservative dual‐criteria found an effect in 33.8% of graphs. Agreement was marginally lower when the objective methods indicated effects (conservative dual‐criteria: *M* = 78%; support vector classifier: *M* = 79%) compared to no effect (conservative dual‐criteria: *M* = 87%; support vector classifier: *M* = 87%). Agreement was lower when the methods indicated an effect compared to no effect for 13 of the 14 comparisons, with the exception of Rater A with the support vector classifier.

Table 3 shows the proportion of graphs with different phase lengths for each of the four groups. When visual raters agreed, most graphs had only three points in Phase A. In contrast, graphs on which visual raters disagreed amongst themselves or with the conservative dual‐criteria method were more likely to have 10 or more points in Phase A. For Phase B, more graphs with three points were present when visual raters agreed, but this difference was offset by the higher proportion of graphs with four or five points in Phase B when visual raters disagreed amongst themselves or with the conservative dual‐criteria method. A more in‐depth analysis of the patterns of agreement and disagreement is available in Supporting Information.

**Table 3:** Proportion of Graphs with Given Phase Lengths in the Presence and Absence of Agreement

		Number of Data Points in the Phase			
		3	4 or 5	6 to 9	10 or more
Phase A		*n* = 96	*n* = 18	*n* = 30	*n* = 54
	Visual inspection – Agreement	.531	.091	.143	.234
	Visual inspection – Disagreement	.130	.087	.217	.565
	CDC – Disagreement	.133	.267	.067	.533
	SVC – Disagreement	.615	.000	.231	.154
Phase B		*n* = 66	*n* = 18	*n* = 42	*n* = 72
	Visual inspection – Agreement	.354	.086	.211	.349
	Visual inspection – Disagreement	.174	.130	.217	.478
	CDC – Disagreement	.067	.267	.400	.267
	SVC – Disagreement	.308	.077	.154	.462

*Note*. The disagreements for the CDC and SVC are relative to exemplars on which visual raters mostly agreed (see text for details). CDC: conservative dual‐criteria method, SVC: support vector classifier.

## Discussion

Our results showed that average agreement was generally consistent across different methods of analysis when analyzing clinical data. That is, the raters, the conservative dual‐criteria method, and the support vector classifier agreed with each other on similar proportions of graphs. Moreover, correlations were high when we asked that the raters provide a score varying from 0 to 10, suggesting that the confidence in their ratings remains generally consistent. This result extends prior research, which has been mostly limited to examining binary classification (i.e., change vs. no change) although there are exceptions (e.g., 100‐point scale, DeProspero & Cohen, 1979). A further analysis indicated that agreement was marginally higher when the objective methods suggested that there was no change in a graph. One potential explanation is that our graphs showing no effect depicted more stable patterns than those showing an effect. Notably, some of the graphs showing no effect showed no change from one phase to the next (i.e., two flat lines of equal level), which facilitated agreement. In general, our findings suggest that agreement between the two objective methods and visual raters is no different than agreement between raters themselves.

The agreements observed in the current study were similar to those reported by Wolfe et al. (2018) using published datasets. This result is promising as it suggests that their published graphs served as an acceptable approximation of clinical graphs, or at least that both types of graphs produce similar ratings. In contrast, raters performed better on the clinical graphs than previously reported by Lanovaz and Hranchuk (2021) on simulated ones. One potential explanation is that simulated graphs may exaggerate patterns (e.g., trend) that are infrequent in clinical graphs, which can be difficult to analyze. Alternatively, clinical graphs may show larger, less ambiguous effects that facilitate analysis and make them easier to rate consistently. Regardless of the cause, this result underlines the importance of replicating studies conducted using simulated data with nonsimulated data.

The support vector classifier did not match visual inspection more closely than the conservative dual‐criteria method, which is inconsistent with results reported by Lanovaz et al. (2020), who examined thesis and dissertation data. This discrepancy may be the result of the Lanovaz et al. procedures that included only two raters. Additionally, having only three points in Phase A was associated with fewer disagreements, which is inconsistent with prior research with simulated data (e.g., Fisher et al., 2003; Lanovaz & Hranchuk, 2021). One potential explanation is that the decisions of behavior analysts are typically response guided. Hence, behavior analysts may stop collecting data early when they observe stability, making the graphs easier to agree on (i.e., less variable).

Prior research has used simulated or published datasets with professors and researchers as raters (e.g., Ford et al., 2020; Lanovaz & Hranchuk, 2021; Wolfe et al., 2016, 2018). We used clinical data with varying phase lengths and trends, containing both effective and ineffective interventions, and with varying degrees of variability, and most raters were practitioners, all of which may extend prior research on the topic. We also used a wide range of variables and metrics (i.e., percentage, latency, responses per minute) from pediatric feeding data with varying characteristics, including extinction bursts, extinction‐induced variability, as well as delayed effects. In pediatric feeding, consistency of baseline replication data may be lower because of graduated exposure and skill development. Clinical cut‐offs and goals must be considered, for example, with latency to swallowing and negative vocalizations. It is important to acknowledge that it is unclear whether and the degree to which our clinical datasets differed from published or simulated datasets in other studies. Given these considerations, visual inspection and objective methods remained comparable with these clinical datasets.

Researchers, professors, and supervisors may use objective‐analysis methods to train visual raters more efficiently and to improve reliability and decrease decision‐making errors. In clinical practice, decisions are made in real time along with data collection. We used machine learning post hoc on clinical data, but future studies could examine its use in real time during the treatment evaluation to aid in decision making and decrease errors. Similar to entering and graphing data into Excel to perform visual inspection during treatment evaluation, practitioners could enter or paste data into an internet‐based application and immediately receive output results inclusive of graphs to assist with decision making in real time. For more advanced applications of machine learning by researchers and professors, tutorials employing free software are already available (e.g., Turgeon & Lanovaz, 2020).

As with most research involving nonsimulated data, the main limitation of our study is that agreement does not equate validity. Two methods may agree, but still produce an incorrect rating. That said, it remains important to examine how agreement varies under naturalistic conditions (i.e., with nonsimulated clinical data). A second limitation is that our dataset was too small (few exemplars) to fully examine dataset characteristics (e.g., phase length, variability, trend, effect size) that may impact results. In the future, researchers should conduct replications with larger datasets to isolate the effects of these variables.

An additional limitation is that participants rated blinded graphs without clinically relevant information (e.g., phase and axis labels indicating the behavior, measurement system, scale, and condition/intervention), reducing the ecological validity of our study. This information can be highly important when making decisions about clinical data. Prior research on the impact of such clinically relevant information on interrater agreement has been shown to be minimal. Ninci et al. (2015) did not identify an association between providing clinically relevant information and agreement. Ford et al. (2020) also did not find that providing such information impacted agreement on published research data, but all variables were equally scaled percentages aimed to increase behavior. An important area of future research is to compare agreement for a variety of graphs rated with and without relevant clinical information. Additional research could examine the impact of specific types of clinical information on interrater agreement. Another extension for future research would be to compare agreement to ratings of graphs made for consecutive phases in sequential order for the entire treatment evaluation, approximating the conditions under which behavior analysts typically analyze single‐case data.

Finally, the study relied on quasiexperimental AB designs for analyses, which limits the applicability of the results. AB designs are insufficient to demonstrate experimental control. Our instructions to the raters asked them to imagine the rest of the graph had the AB graph been replicated, producing an ABAB design. The hypothetical instruction (i.e., to decide on functional control as if there were a reversal and replication) removed the real‐life variables one may observe in practice. Visual raters analyzing a graph as a whole (rather than as independent AB graphs) may produce different conclusions regarding the presence of functional control. We do not yet know if the machine‐learning outcomes would have differed if ABAB graphs were used. We took this approach because AB comparisons serve as the basic unit of many other types of graphs (i.e., multiple baseline, reversal, and changing‐criterion designs), and we did not have enough complete ABAB graphs to conduct our analyses. Additionally, the use of AB comparisons is not unique to machine learning and is also used by all other proposed objective methods designed to supplement visual inspection (e.g., Fisher et al., 2003; Manolov & Vannest, 2019). However, it is possible to develop machine learning models to analyze the full range of single‐case experimental designs, which is an important future research direction. As Wolfe et al. (2018) focused on multiple baseline designs, future research should address these issues carefully by replicating their study with other types of experimental designs. Nonetheless, our study clearly supports prior research by showing that structured methods of analyses may produce results generally consistent with visual inspection.

## Supporting information


**Appendix S1** Supporting Information

## Footnotes

^1^
A set of instructions designed to solve a general, but well‐specified, problem (Skiena, 2008)
