# Supplementary material for: Agreement between visual inspection and objective analysis methods: A replication and extension
Source: J Appl Behav Anal. 2022 Apr 27;55(3):986–96. doi: 10.1002/jaba.921 (PMC9323513; doi:10.1002/jaba.921)
Supplement: Supplementary file 1 — Appendix S1 Supporting Information [file JABA-55-986-s001.docx]

**Supporting Information**

**Agreement Between Visual Inspection and Objective Analysis Methods:**

**A Replication and Extension**

For each group, we computed and compared the standardized mean difference, the autocorrelation, as well as the slopes of Phases A and B. The standardized mean difference consisted of subtracting the mean of Phase B from the mean of Phase A and dividing the difference by pooled standard deviation of the two phases. To compute the autocorrelation, we used the same procedures as previously described by Shadish and Sullivan (2011). That is, our analyses extracted the residuals from a linear regression of the points on treatment, time, and the interaction between the two. Then, we computed the lag 1 correlation on these residuals. For the slope, our code instructed the program to extract the standardized slope coefficient of a linear regression for points on time. When a reduction in behavior was expected in a graph, we multiplied the standardized mean difference and standardized slope values by -1 so that negative values always represented patterns in the opposite direction of the expected change. These standardizations were essential as the different clinical measures had widely different scales.

Figure A1 shows the distributions of standardized mean differences and autocorrelations across each group. When visual raters agreed, the median value of the standardized mean difference (upper panel) was lower than when there was disagreement, and approximately 90% of values fell between -1 and 4. In comparison, the standardized mean differences tended to range between 0 and 2 for disagreements. For this analysis, the support vector classifier had the highest median and lowest range. For autocorrelation (lower panel), we did not observe consistent differences across groups, but we found generally negative autocorrelations overall.

Figure A2 displays boxplots for the slope. Overall, slopes in Phase A (upper panel) were generally nil or in the opposite direction of the expected change. Contrarily, slopes in Phase B (lower panel) tended to be in the direction of the expected change. Both the visual raters and the conservative dual-criteria method disagreed more frequently on graphs that showed a negative slope for Phase A. For the slope of Phase B, the disagreement with the conservative dual-criteria group contains a larger proportion of graphs with steep positive slopes than the other groups.

**Reference**

Shadish, W. R., & Sullivan, K. J. (2011). Characteristics of single-case designs used to assess intervention effects in 2008. *Behavior Research Methods*, *43*(4), 971-980. https://doi.org/10.3758/s13428-011-0111-y

**Figure A1**

*Boxplots of the Distribution of Standardized Mean Difference and Autocorrelation of Graphs in the Presence or Absence of Disagreement*


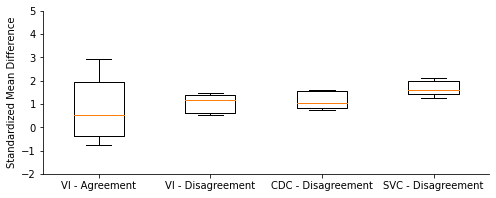

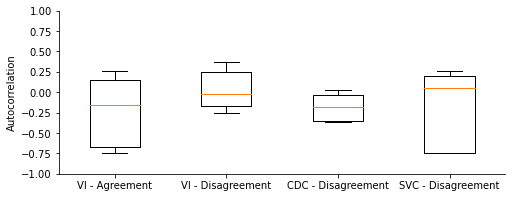


*Note.* The disagreements for the CDC and SVC are relative to exemplars on which visual raters mostly agreed. The whiskers are located at the 15^th^ and 85^th^ percentile ranks. VI: Visual inspection, CDC: conservative dual-criteria method, SVC: support vector classifier

**Figure A2**

*Boxplots of the Distribution of Standardized Slopes for Phases A and B of Graphs in the Presence or Absence of Disagreement*


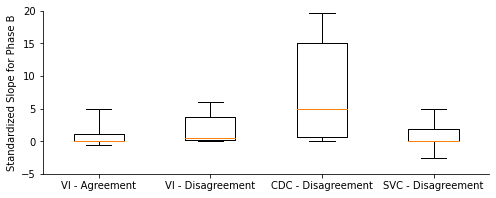

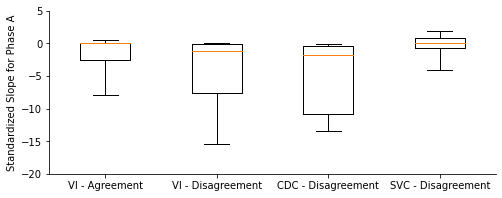


*Note.* The disagreements for the CDC and SVC are relative to exemplars on which visual raters mostly agreed. The whiskers are located at the 15^th^ and 85^th^ percentile ranks. VI: Visual inspection, CDC: conservative dual-criteria method, SVC: support vector classifier
